# A Non-Resolving “Hematoma” Diagnosed as an Arteriovenous Malformation by POCUS

**DOI:** 10.24908/pocus.v7i2.15889

**Published:** 2022-11-21

**Authors:** Michael Freilich, Benjamin Galen, Deborah Schwartz

**Affiliations:** 1 Department of Internal Medicine, Moses-Weiler Residency Training Program, Albert Einstein College of Medicine and Montefiore Medical Center Bronx, NY; 2 Department of Internal Medicine, Division of Hospital Medicine, Albert Einstein College of Medicine and Montefiore Medical Center Bronx, NY

**Keywords:** POCUS, AVM

## Abstract

Point-of-care ultrasound (POCUS) is a useful tool for the evaluation of soft tissue masses. We present the case of a patient with a mass on his forehead initially thought to be a slowly resolving hematoma. POCUS examination of the mass revealed a vascular structure more consistent with a post-traumatic arteriovenous malformation (AVM). This case illustrates how POCUS can be utilized to rapidly assess soft tissue masses and even identify unexpected vascularity.

## Case Report

**Figure 1:**
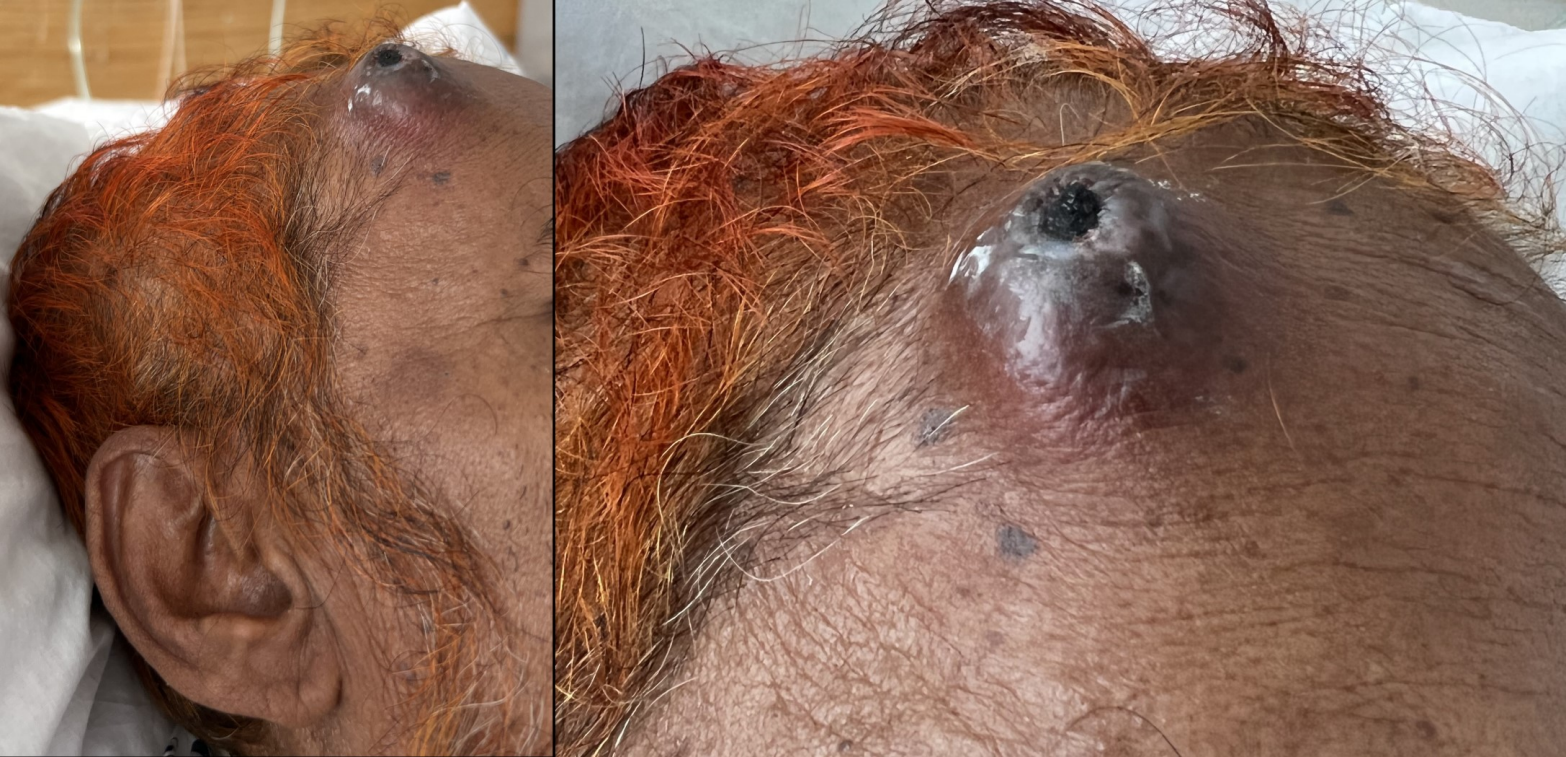
Images of right forehead soft tissue mass.

A 59-year-old gentleman with end stage renal disease, coronary artery disease, type 2 diabetes, and hypertension was admitted to the hospital for dyspnea due to mild volume overload. On physical exam, he was noted to have a 3 x 3 cm soft tissue mass on the right forehead with superficial ulceration (Figure 1). His medication list did not contain any anti-platelet agents or anticoagulation. The patient described a hematoma since a mechanical fall while walking on the street 6 weeks prior with impact to his forehead. He had developed swelling immediately after the fall but did not lose consciousness. He presented to the emergency department (ED) at that time where CT head was performed showing a 3 cm prominent right prefrontal soft tissue hematoma. A needle aspiration was performed with reportedly over half of the fluid in the hematoma drained. He was discharged from the ED. Given increased swelling and pain at the site of the hematoma, he returned to the ED 1 week later where the soft tissue swelling was noted to be 4 cm. Given persistent pain, a repeat needle aspiration was performed and 7 ml of blood was removed. Incision and drainage was also performed with clotted blood removed and he was discharged. Fluid specimens were not sent for laboratory analysis at either visit. 

**Figure 2:**
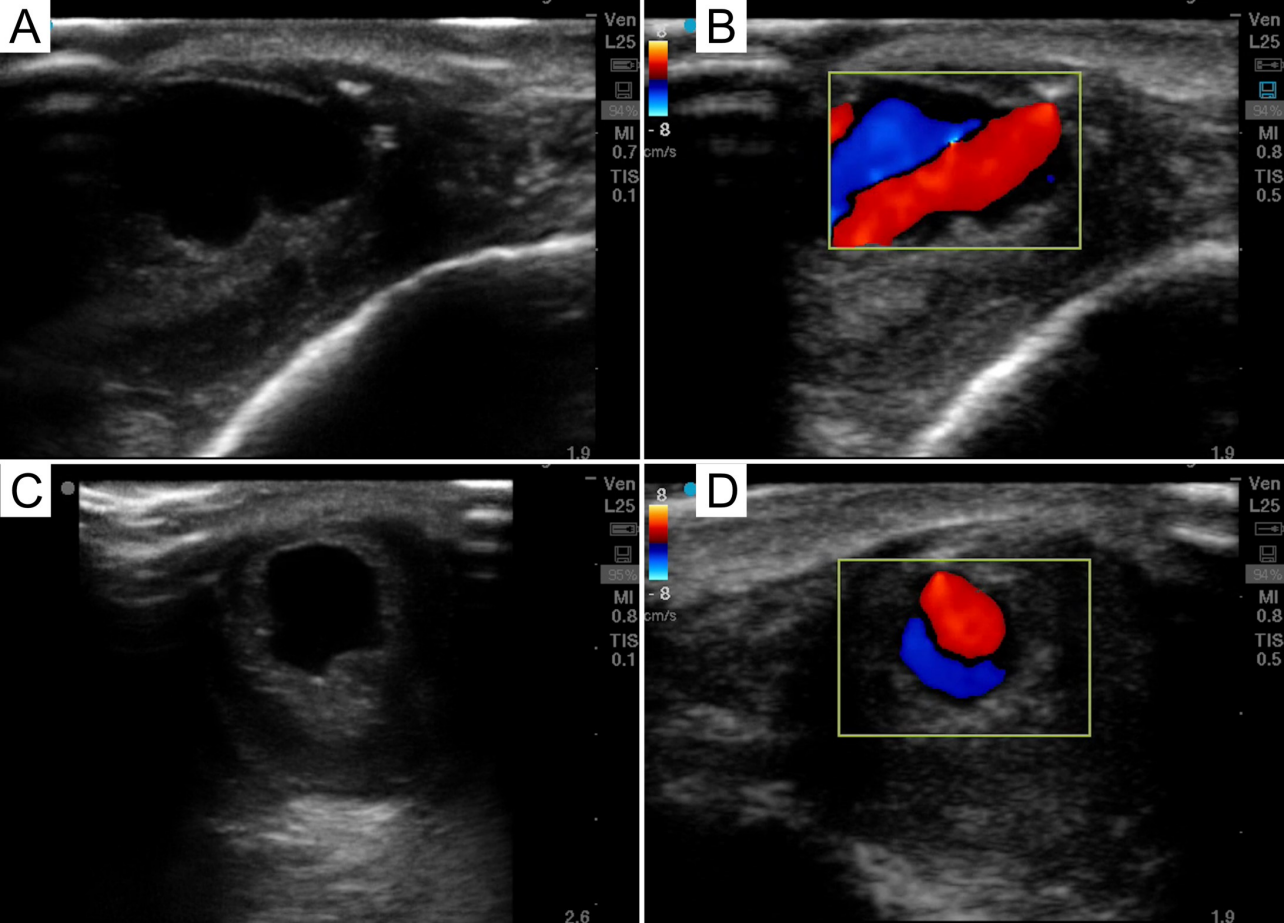
A, B are long axis POCUS images of the right forehead soft tissue mass (B with color Doppler). C, D are short axis POCUS images (D with color Doppler). B and D demonstrate the“Ying-Yang” or “Pepsi” sign, which is due to bidirectional or turbulent blood flow.

**Figure 3:**
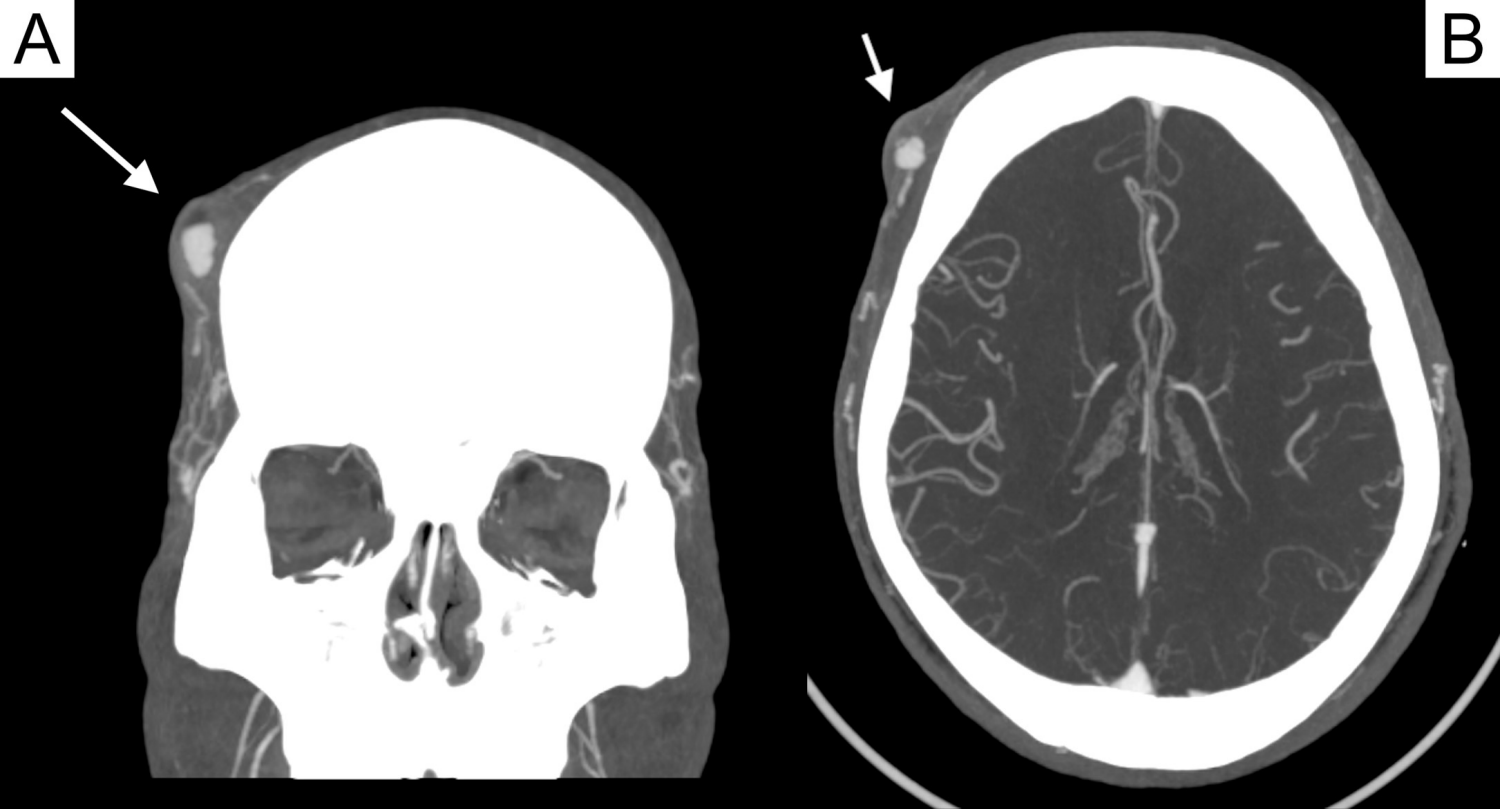
Coronal (A) and Axial (B) Head CT-Angiogram with 7mm round pool of contrast (white arrows) supplied by an artery with a questionable draining vein, consistent with an AVM or less likely pseudoaneurysm.

Since the second visit to the ED, the patient reported that the “hematoma” had recurred status-post aspiration and drainage but was smaller than it was initially and remained painful. Plastic surgery was consulted who noted palpable thrill at lateral aspect of the mass. On POCUS examination, the forehead mass was found to have pulsatile internal flow concerning for a vascularized structure such as an arteriovenous malformation (AVM) (Figure 2 and Supplemental Video S1). CTA was performed which revealed a 7 mm round pool of contrast at the lesion, supplied by an artery with a questionable draining vein, consistent with an AVM or less likely pseudoaneurysm (Figure 3). Given ongoing pain at the site of the soft tissue mass, the patient was planned for outpatient resection of the vascularized soft tissue mass with interventional radiology support. 

## Discussion

A superficial soft tissue mass has a broad differential diagnosis which includes lipoma and other tumors, cyst, hematoma, and atypical abscess. Given the inciting trauma and known antecedent hematoma, a slowly resorbing or persistent hematoma was considered possible in this case, but the duration of the mass for >4 weeks would have been very unusual. Lipomas are subcutaneous collections of adipose tissue commonly occurring in the head and neck region. They have been found to occur after trauma but are usually slow growing  1. An abscess can be considered as a sequela of an infected hematoma; however, this patient never presented with any signs of systemic infection (fever, chills, elevated WBC count). Even less likely in this case was a malignant soft tissue tumor or distal metastasis, as the growth was rapid and there was no history of solid tumor malignancy, respectively. 

POCUS can be helpful in the initial evaluation of a soft tissue mass as it can be used to distinguish some of the characteristic features of this broad differential diagnosis. For lipomas, one would expect to see a lack of blood flow on color Doppler with minimal or no growth on serial measurements using US 2. While the shape and echogenicity of abscesses are variable, mobility of echogenic internal fluid contents when placing slight pressure with the ultrasound probe could lead to a higher clinical suspicion of abscess. Furthermore, surrounding edema in a cobblestone appearance can be seen secondary to the host inflammatory response 3. Malignant soft tissue masses will grow on serial US measurements and are expected to be larger in nature when compared to other diagnoses one may be considering (>4.6 cm). Other common features of malignant masses include irregular borders, deep infiltration through surrounding tissue planes or structures, and increased vascularity on color Doppler 3, 4, 5. Specific to vascular malformations, there were POCUS findings that this patent’s mass exhibited that helped narrow the differential: The “Ying-Yang” or “Pepsi” sign on color Doppler is due to bidirectional or turbulent flow (Figure 2B, 2D). This has been reported in pseudoaneurysms but was seen in this case of suspected post-traumatic AVM 6. 

AV Malformations (AVM) are fistulas between arteries and veins with an incomplete or absent capillary bed in between 7. While most of AVMs are congenital, thought to be related to a lack of apoptosis of primitive arteriovenous shunts, a subset of these malformations can be related to trauma. Trauma is thought to cause a pro-inflammatory state, leading to the development of AVMs 8. An AVM should be suspected when a careful physical exam reveals pulsatility or thrill to the mass or when auscultation of the area reveals a bruit. 

A vascular imaging modality is necessary to diagnose an AVM and assist in treatment decision making. Formal vascular ultrasound is usually the first imaging modality used due to its ability to be performed at the bedside, low cost, and no radiation or contrast exposure. Ultrasound is limited in its ability to determine depth of invasion into surrounding structures. MRI or CT-angiogram provides better spatial context along with hemodynamic information. The gold standard in diagnosis is a catheter based digital-subtraction angiogram 9. Angiography distinctly shows the vasculature and allows for therapeutic guidance based off the resulting images. 

Once a soft tissue mass is diagnosed as an AVM, early intervention through a multidisciplinary approach is best. AVM can be managed conservatively if it is asymptomatic with no distal ischemia or compression. Surgical treatment options include embolization, coiling, resection and transcutaneous scleropathy.

POCUS is an essential tool to rapidly evaluate a soft tissue mass. As in the case presented, significant vascularity or arterial flow in a soft tissue mass should raise immediate concern for an AVM or pseudoaneurysm. 

## Patient Consent

The patient provided written consent for presentation of his case. 

## Disclosures

None.

## Supplementary Material

 Video S1 The top two POCUS clips are long axis images of a right forehead soft tissue mass. The bottom two clips are short axis of this structure. The clips on the right use color Doppler and demonstrate pulsatile arterial flow inside this lesion, consistent with an AVM.
